# Supplementary material for: A Noble Extract of Pseudomonas sp. M20A4R8 Efficiently Controlling the Influenza Virus-Induced Cell Death
Source: Microorganisms. 2024 Mar 28;12(4):677. doi: 10.3390/microorganisms12040677 (PMC11051866; doi:10.3390/microorganisms12040677)
Supplement: Supplementary file 1 [file microorganisms-12-00677-s001.zip › microorganisms-2924133-supplementary.pdf]

# *Supplementary Materials*

## Supplementary Figures

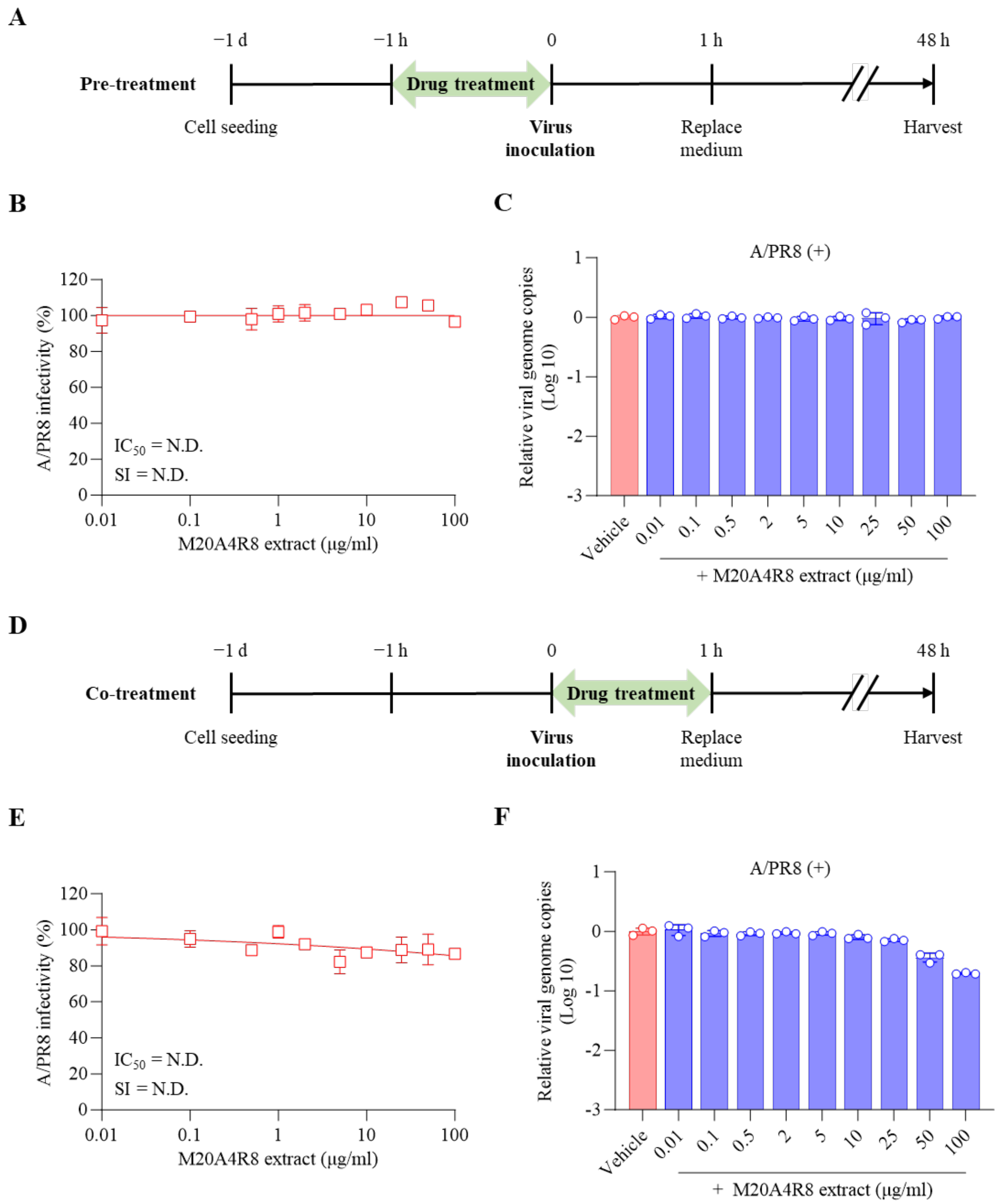


**Supplementary Figure S1.** Antiviral effect of M20A4R8 extract by pre-treatment (A–C) and co-treatment (D–F) against A/PR8 strain in MDCK cell. (A) Schematic diagram of virus inoculation and extract treatment. (B) $IC_{50}$ and SI of M20A4R8 extract measured by CPE-inhibition assay. (C) Viral genome copies detected by RT-qPCR. (D) Schematic diagram of virus inoculation and extract treatment. (E) $IC_{50}$ and SI of M20A4R8 extract measured by CPE-inhibition assay. (F) Viral genome copies detected by RT-qPCR. All data in the graphs are presented as arithmetic means ± S.D. from 3 independent experiments. N.D., not determined

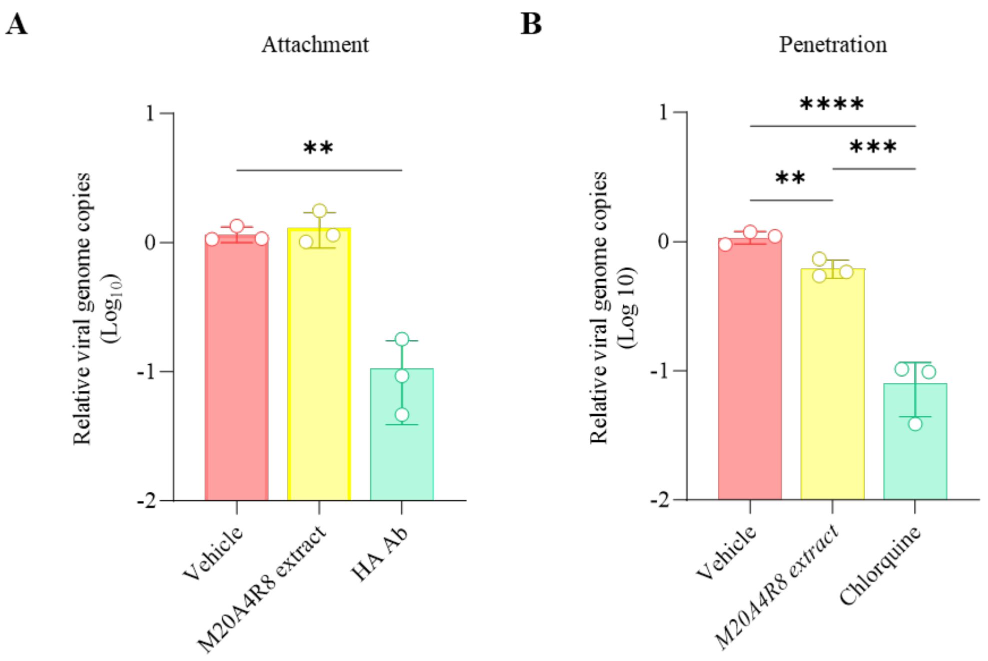


**Supplementary FigureS2.** Viral genome copies measured at 20 h of incubation after virus attachment and penetration assays along with the treatment of M20A4R8 extract (50 μg/mL). (A) The extract was pre-tre ated before the viral binding to the MDCK cells at 4 °C for 1 h, followed by infection with A/PR8 strai n (1 MOI). (B) After binding the A/PR8 strain (1 MOI) to the host cells, the extract was absorbed at 37 ° C for 1 h. Viral genome copies were measured by RT-qPCR using a pair of primer-detecting IAV PB1. All data in the graphs are presented as arithmetic means ± S.D. from 3 independent experiments. HA, h emagglutinin. **, *p* < 0.01; ****, *p* < 0.0001.
